# 1-(Isopropyl­amino)-3-phen­oxy­propan-2-ol

**DOI:** 10.1107/S1600536812000566

**Published:** 2012-01-11

**Authors:** Xuehui Hou, Zigang Li, Quanjian Lv

**Affiliations:** a Department of Quality Detection and Management, Zhengzhou College of Animal Husbandry Engineering, Zhengzhou 450011, People’s Republic of China

## Abstract

In the crystal structure of the title amino alcohol derivitive, C_12_H_19_NO_2_, mol­ecules are linked by N—H⋯O hydrogen bonds. The mol­ecular structure exhibits an intra­molecular O—H⋯N hydrogen bond.

## Related literature

For applications of amino alcohols and their derivatives, see: Ellison *et al.* (2005 ▶); Li *et al.* (2004 ▶).
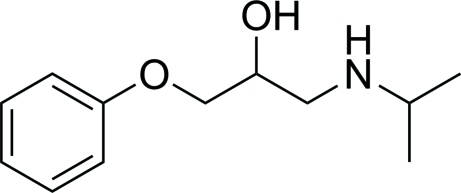


## Experimental

### 

#### Crystal data

C_12_H_19_NO_2_
*M* *_r_* = 209.28
Tetragonal, 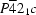

*a* = 15.1162 (17) Å
*c* = 10.9448 (14) Å
*V* = 2500.9 (5) Å^3^
*Z* = 8
Mo *K*α radiation
μ = 0.08 mm^−1^
*T* = 298 K
0.45 × 0.38 × 0.37 mm

#### Data collection

Bruker SMART CCD diffractometer
Absorption correction: multi-scan (*SADABS*; Sheldrick, 1996 ▶) *T*_min_ = 0.967, *T*_max_ = 0.973
9624 measured reflections
1252 independent reflections
676 reflections with *I* > 2σ(*I*)
*R*_int_ = 0.125

#### Refinement

*R*[*F* ^2^ > 2σ(*F* ^2^)] = 0.064
*wR*(*F* ^2^) = 0.221
*S* = 1.16
1252 reflections
138 parameters
16 restraints
H-atom parameters constrained
Δρ_max_ = 0.24 e Å^−3^
Δρ_min_ = −0.25 e Å^−3^

### 

Data collection: *SMART* (Siemens, 1996 ▶); cell refinement: *SAINT* (Siemens, 1996 ▶); data reduction: *SAINT*; program(s) used to solve structure: *SHELXS97* (Sheldrick, 2008 ▶); program(s) used to refine structure: *SHELXL97* (Sheldrick, 2008 ▶); molecular graphics: *SHELXTL* (Sheldrick, 2008 ▶) and *DIAMOND* (Brandenburg, 1998 ▶); software used to prepare material for publication: *SHELXTL*.

## Supplementary Material

Crystal structure: contains datablock(s) I, global. DOI: 10.1107/S1600536812000566/lx2213sup1.cif

Structure factors: contains datablock(s) I. DOI: 10.1107/S1600536812000566/lx2213Isup2.hkl

Supplementary material file. DOI: 10.1107/S1600536812000566/lx2213Isup3.cml

Additional supplementary materials:  crystallographic information; 3D view; checkCIF report

## Figures and Tables

**Table 1:** Hydrogen-bond geometry (Å, °)

*D*—H⋯*A*	*D*—H	H⋯*A*	*D*⋯*A*	*D*—H⋯*A*
O2—H2⋯N1	0.82	2.31	2.760 (7)	115
N1—H1⋯O2^i^	0.90	1.84	2.742 (7)	179

Symmetry code: (i) 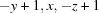.

**Fig. 1.**
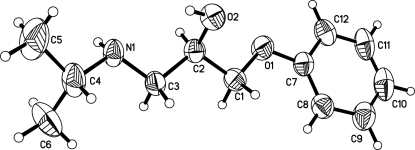
The molecular structure of the title compound with the atom numbering scheme. Displacement ellipsoids are drawn at the 50% probability level. H atoms are presented as small spheres of arbitrary radius.

**Fig. 2.**
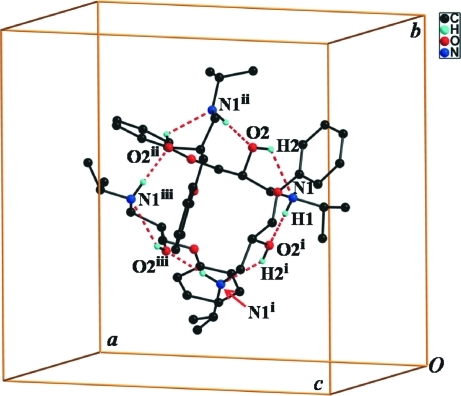
A view of the N—H···O and O—H···N hydrogen bonds (dotted lines) in the crystal structure of the title compound. [Symmetry codes: (i) - y + 1, x, - z + 1 (ii) y, - x + 1, - z + 1 (iii) - x + 1, - y + 1, z. ]

## supplementary crystallographic information

### Comment

Amino alcohols are important structural elements for asymmetric catalysis(Li *et al.*, 2004) as well as in biologically active compounds(Ellison *et al.*, 2005). In order to develop new applications for amino alcohols and their derivatives, structural modifications of these compounds have been extensively investigated. As a contribution in this filed, we report here the crystal structure of the title compound.

The molecular structure of the title compound is shown in Fig.1.

The title compound crystallizes as the non–centrosymmetric space group *P*_-421_*_c_* in spite of having no asymmetric C atoms.

The crystal packing (Fig. 2) is stabilized by intermolecular N—H···O hydrogen bonds (see, Table 1; second entry). The crystal packing (Fig. 2) is further stabilized by intramolecular O—H···N hydrogen bonds (see, Table 1; first entry).

### Experimental

To a solution of 2-(phenoxymethyl)oxirane (15.0 g, 0.1 mol) in acetone (200 ml), propan–2–amine (86.7 ml, 1.0 mol) was added. The mixture was stirred at room temperature for 6 h, followed by concentrated under reduced pressure and purification by crystallization from ethyl acetate, giving title compound as colourless single crystals suitabl for X-1ray analysis.

### Refinement

All the Friedel pairs were merged. All H atoms were placed geometrically and treated as riding on their parent atoms, with C—H = 0.93–0.96 Å and *U*_iso_(H) = 1.2*U*_eq_(C) or 1.5*U*_eq_(C) for methyl H atoms.

### Crystal data

**Table:**

C_12_H_19_NO_2_	*D*_x_ = 1.112 Mg m^−^^3^
*M**_r_* = 209.28	Mo *K*α radiation, λ = 0.71073 Å
Tetragonal, *P*42_1_*c*	Cell parameters from 2006 reflections
Hall symbol: P -4 2n	θ = 2.3–19.8°
*a* = 15.1162 (17) Å	µ = 0.08 mm^−^^1^
*c* = 10.9448 (14) Å	*T* = 298 K
*V* = 2500.9 (5) Å^3^	Block, colourless
*Z* = 8	0.45 × 0.38 × 0.37 mm
*F*(000) = 912	

### Data collection

**Table:**

Bruker SMART CCD diffractometer	1252 independent reflections
Radiation source: fine-focus sealed tube	676 reflections with *I* > 2σ(*I*)
graphite	*R*_int_ = 0.125
Detector resolution: 10.0 pixels mm^-1^	θ_max_ = 25.0°, θ_min_ = 1.9°
φ and ω scans	*h* = −17→17
Absorption correction: multi-scan (*SADABS*; Sheldrick, 1996)	*k* = −17→9
*T*_min_ = 0.967, *T*_max_ = 0.973	*l* = −7→13
9624 measured reflections	

### Refinement

**Table:**

Refinement on *F*^2^	Primary atom site location: structure-invariant direct methods
Least-squares matrix: full	Secondary atom site location: difference Fourier map
*R*[*F*^2^ > 2σ(*F*^2^)] = 0.064	Hydrogen site location: difference Fourier map
*wR*(*F*^2^) = 0.221	H-atom parameters constrained
*S* = 1.16	*w* = 1/[σ^2^(*F*_o_^2^) + (0.0662*P*)^2^ + 2.183*P*] where *P* = (*F*_o_^2^ + 2*F*_c_^2^)/3
1252 reflections	(Δ/σ)_max_ < 0.001
138 parameters	Δρ_max_ = 0.24 e Å^−^^3^
16 restraints	Δρ_min_ = −0.25 e Å^−^^3^

### Special details

**Table:**

Geometry. All esds (except the esd in the dihedral angle between two l.s. planes) are estimated using the full covariance matrix. The cell esds are taken into account individually in the estimation of esds in distances, angles and torsion angles; correlations between esds in cell parameters are only used when they are defined by crystal symmetry. An approximate (isotropic) treatment of cell esds is used for estimating esds involving l.s. planes.
Refinement. Refinement of F^2^ against ALL reflections. The weighted R-factor wR and goodness of fit S are based on F^2^, conventional R-factors R are based on F, with F set to zero for negative F^2^. The threshold expression of F^2^ > 2sigma(F^2^) is used only for calculating R-factors(gt) etc. and is not relevant to the choice of reflections for refinement. R-factors based on F^2^ are statistically about twice as large as those based on F, and R- factors based on ALL data will be even larger.

### Fractional atomic coordinates and isotropic or equivalent isotropic displacement parameters (Å^2^)

**Table:**

	*x*	*y*	*z*	*U*_iso_*/*U*_eq_	
O1	0.5032 (3)	0.6282 (4)	0.7225 (4)	0.0824 (15)	
O2	0.3665 (3)	0.6526 (3)	0.5499 (4)	0.0750 (14)	
H2	0.3250	0.6451	0.5027	0.090*	
N1	0.2523 (3)	0.5115 (4)	0.5235 (5)	0.0700 (17)	
H1	0.2831	0.4635	0.4999	0.084*	
C1	0.4135 (5)	0.6047 (5)	0.7440 (7)	0.074 (2)	
H1C	0.3813	0.6547	0.7774	0.089*	
H1D	0.4101	0.5561	0.8016	0.089*	
C2	0.3755 (5)	0.5783 (5)	0.6253 (7)	0.0639 (17)	
H2A	0.4151	0.5357	0.5858	0.077*	
C3	0.2859 (4)	0.5375 (5)	0.6394 (7)	0.0650 (19)	
H3A	0.2898	0.4863	0.6925	0.078*	
H3B	0.2459	0.5798	0.6768	0.078*	
C4	0.1582 (5)	0.4884 (6)	0.5218 (7)	0.099 (3)	
H4	0.1242	0.5369	0.5583	0.119*	
C5	0.1313 (6)	0.4781 (9)	0.3916 (9)	0.145 (5)	
H5A	0.1431	0.5319	0.3481	0.218*	
H5B	0.0692	0.4650	0.3874	0.218*	
H5C	0.1642	0.4305	0.3553	0.218*	
C6	0.1420 (7)	0.4060 (7)	0.5930 (11)	0.152 (5)	
H6A	0.1765	0.3586	0.5592	0.228*	
H6B	0.0804	0.3909	0.5890	0.228*	
H6C	0.1588	0.4152	0.6766	0.228*	
C7	0.5482 (4)	0.6682 (5)	0.8129 (7)	0.072 (2)	
C8	0.5118 (5)	0.6915 (5)	0.9248 (6)	0.083 (2)	
H8	0.4530	0.6790	0.9430	0.100*	
C9	0.5660 (6)	0.7339 (5)	1.0086 (8)	0.099 (3)	
H9	0.5437	0.7495	1.0848	0.119*	
C10	0.6534 (6)	0.7533 (6)	0.9800 (11)	0.111 (4)	
H10	0.6886	0.7831	1.0362	0.133*	
C11	0.6867 (7)	0.7298 (5)	0.8730 (10)	0.103 (3)	
H11	0.7458	0.7418	0.8563	0.124*	
C12	0.6357 (4)	0.6876 (5)	0.7852 (8)	0.086 (2)	
H12	0.6596	0.6727	0.7096	0.103*	

### Atomic displacement parameters (Å^2^)

**Table:**

	*U*^11^	*U*^22^	*U*^33^	*U*^12^	*U*^13^	*U*^23^
O1	0.056 (3)	0.109 (4)	0.082 (3)	−0.016 (3)	−0.003 (3)	−0.020 (3)
O2	0.067 (3)	0.077 (3)	0.081 (3)	0.001 (3)	−0.007 (3)	0.005 (3)
N1	0.048 (3)	0.070 (4)	0.092 (4)	−0.002 (3)	−0.002 (3)	−0.025 (4)
C1	0.056 (4)	0.088 (5)	0.078 (5)	−0.013 (4)	0.005 (4)	−0.009 (4)
C2	0.056 (4)	0.064 (4)	0.072 (4)	0.001 (3)	−0.005 (4)	−0.003 (4)
C3	0.057 (4)	0.062 (4)	0.076 (5)	−0.008 (3)	0.001 (4)	−0.009 (4)
C4	0.048 (4)	0.110 (7)	0.139 (8)	−0.012 (4)	−0.004 (5)	−0.040 (7)
C5	0.075 (6)	0.211 (13)	0.150 (9)	−0.007 (8)	−0.041 (7)	−0.060 (9)
C6	0.102 (8)	0.157 (10)	0.196 (13)	−0.066 (7)	0.020 (8)	−0.001 (10)
C7	0.067 (5)	0.066 (5)	0.082 (5)	−0.004 (4)	−0.011 (4)	−0.002 (4)
C8	0.089 (6)	0.080 (5)	0.080 (5)	−0.011 (5)	−0.015 (5)	0.012 (5)
C9	0.125 (8)	0.079 (6)	0.093 (6)	−0.005 (6)	−0.030 (6)	−0.001 (5)
C10	0.129 (10)	0.077 (6)	0.127 (9)	−0.017 (6)	−0.051 (8)	−0.007 (6)
C11	0.091 (7)	0.078 (6)	0.141 (9)	−0.024 (5)	−0.042 (7)	−0.001 (7)
C12	0.065 (5)	0.081 (5)	0.113 (6)	−0.011 (4)	−0.011 (5)	0.003 (5)

### Geometric parameters (Å, °)

**Table:**

O1—C7	1.344 (8)	C5—H5A	0.9600
O1—C1	1.422 (8)	C5—H5B	0.9600
O2—C2	1.400 (8)	C5—H5C	0.9600
O2—H2	0.8200	C6—H6A	0.9600
N1—C3	1.422 (9)	C6—H6B	0.9600
N1—C4	1.464 (9)	C6—H6C	0.9600
N1—H1	0.9000	C7—C8	1.388 (2)
C1—C2	1.475 (9)	C7—C12	1.388 (2)
C1—H1C	0.9700	C8—C9	1.388 (2)
C1—H1D	0.9700	C8—H8	0.9300
C2—C3	1.497 (9)	C9—C10	1.388 (2)
C2—H2A	0.9800	C9—H9	0.9300
C3—H3A	0.9700	C10—C11	1.323 (13)
C3—H3B	0.9700	C10—H10	0.9300
C4—C5	1.490 (8)	C11—C12	1.388 (2)
C4—C6	1.490 (8)	C11—H11	0.9300
C4—H4	0.9800	C12—H12	0.9300
			
C7—O1—C1	118.3 (6)	C4—C5—H5B	109.5
C2—O2—H2	109.5	H5A—C5—H5B	109.5
C3—N1—C4	115.1 (6)	C4—C5—H5C	109.5
C3—N1—H1	107.1	H5A—C5—H5C	109.5
C4—N1—H1	107.9	H5B—C5—H5C	109.5
O1—C1—C2	107.1 (6)	C4—C6—H6A	109.5
O1—C1—H1C	110.3	C4—C6—H6B	109.5
C2—C1—H1C	110.3	H6A—C6—H6B	109.5
O1—C1—H1D	110.3	C4—C6—H6C	109.5
C2—C1—H1D	110.3	H6A—C6—H6C	109.5
H1C—C1—H1D	108.6	H6B—C6—H6C	109.5
O2—C2—C1	109.9 (6)	O1—C7—C8	124.3 (6)
O2—C2—C3	107.7 (6)	O1—C7—C12	114.6 (6)
C1—C2—C3	111.9 (6)	C8—C7—C12	121.1 (7)
O2—C2—H2A	109.1	C7—C8—C9	117.8 (7)
C1—C2—H2A	109.1	C7—C8—H8	121.1
C3—C2—H2A	109.1	C9—C8—H8	121.1
N1—C3—C2	110.2 (6)	C10—C9—C8	120.6 (8)
N1—C3—H3A	109.6	C10—C9—H9	119.7
C2—C3—H3A	109.6	C8—C9—H9	119.7
N1—C3—H3B	109.6	C11—C10—C9	120.4 (10)
C2—C3—H3B	109.6	C11—C10—H10	119.8
H3A—C3—H3B	108.1	C9—C10—H10	119.8
N1—C4—C5	107.6 (7)	C10—C11—C12	121.7 (10)
N1—C4—C6	110.6 (7)	C10—C11—H11	119.2
C5—C4—C6	111.5 (10)	C12—C11—H11	119.2
N1—C4—H4	109.0	C11—C12—C7	118.4 (8)
C5—C4—H4	109.0	C11—C12—H12	120.8
C6—C4—H4	109.0	C7—C12—H12	120.8
C4—C5—H5A	109.5		
			
C7—O1—C1—C2	169.4 (6)	C1—O1—C7—C12	179.0 (7)
O1—C1—C2—O2	−70.2 (8)	O1—C7—C8—C9	−178.9 (7)
O1—C1—C2—C3	170.2 (6)	C12—C7—C8—C9	−0.6 (12)
C4—N1—C3—C2	−167.4 (6)	C7—C8—C9—C10	0.9 (13)
O2—C2—C3—N1	60.3 (7)	C8—C9—C10—C11	−1.5 (15)
C1—C2—C3—N1	−178.9 (7)	C9—C10—C11—C12	1.9 (16)
C3—N1—C4—C5	170.6 (8)	C10—C11—C12—C7	−1.6 (14)
C3—N1—C4—C6	−67.4 (10)	O1—C7—C12—C11	179.4 (7)
C1—O1—C7—C8	−2.6 (11)	C8—C7—C12—C11	0.9 (12)

### Hydrogen-bond geometry (Å, °)

**Table:**

*D*—H···*A*	*D*—H	H···*A*	*D*···*A*	*D*—H···*A*
O2—H2···N1	0.82	2.31	2.760 (7)	115
N1—H1···O2^i^	0.90	1.84	2.742 (7)	179

Symmetry codes: (i) −*y*+1, *x*, −*z*+1.

## References

[bb1] Brandenburg, K. (1998). *DIAMOND* Crystal Impact GbR, Bonn, Germany.

[bb2] Ellison, K. E. & Gandhi, G. (2005). *Drugs*, pp. 787–797. 10.2165/00003495-200565060-00006 15819591

[bb3] Li, Y., He, B., Qin, B., Feng, X. M. & Zhang, G. L. (2004). *J. Org. Chem.* pp. 7910–7913. 10.1021/jo0488356 15527269

[bb4] Sheldrick, G. M. (1996). *SADABS* University of Göttingen, Germany.

[bb5] Sheldrick, G. M. (2008). *Acta Cryst.* A**64**, 112–122. 10.1107/S0108767307043930 18156677

[bb6] Siemens (1996). *SAINT* and *SMART* Siemens Analytical X-ray Instruments Inc., Madison, Wisconsin, USA.

